# Evaluation of a Hybrid Dynamic Stabilization and Fusion System in the Lumbar Spine: A 10 Year Experience

**DOI:** 10.7759/cureus.637

**Published:** 2016-06-10

**Authors:** Ahmed Kashkoush, Nitin Agarwal, Erin Paschel, Ezequiel Goldschmidt, Peter C Gerszten

**Affiliations:** 1 Department of Neurological Surgery, University of Pittsburgh Medical Center

**Keywords:** adjacent-segment disease, dynamic stabilization, dynesys-transition-optima, lumbar spinal fusion, motion preservation, degenerative disc disease

## Abstract

Introduction: The development of adjacent-segment disease is a recognized consequence of lumbar fusion surgery. Posterior dynamic stabilization, or motion preservation, techniques have been developed which theoretically decrease stress on adjacent segments following fusion. This study presents the experience of using a hybrid dynamic stabilization and fusion construct for degenerative lumbar spine pathology in place of rigid arthrodesis.

Methods: A clinical cohort investigation was conducted of 66 consecutive patients (31 female, 35 male; mean age: 53 years, range: 25 – 76 years) who underwent posterior lumbar instrumentation with the Dynesys Transition Optima (DTO) implant (Zimmer-Biomet Spine, Warsaw, IN) hybrid dynamic stabilization and fusion system over a 10-year period. The median length of follow-up was five years. DTO consists of pedicle screw fixation coupled to a rigid rod as well as a flexible longitudinal connecting system. All patients had symptoms of back pain and neurogenic claudication refractory to non-surgical treatment. Patients underwent lumbar arthrodesis surgery in which the hybrid system was used for stabilization instead of arthrodesis of the stenotic adjacent level.

Results: Indications for DTO instrumentation were primary degenerative disc disease (n = 52) and failed back surgery syndrome (n = 14). The most common dynamically stabilized and fused segments were L3-L4 (n = 37) and L5-S1 (n = 33), respectively. Thirty-eight patients (56%) underwent decompression at the dynamically stabilized level, and 57 patients (86%) had an interbody device placed at the level of arthrodesis. Complications during the follow-up period included a single case of screw breakage and a single case of pseudoarthrosis. Ten patients (15%) subsequently underwent conversion of the dynamic stabilization portion of their DTO instrumentation to rigid spinal arthrodesis.

Conclusion: The DTO system represents a novel hybrid dynamic stabilization and fusion construct. This 10-year experience found the device to be highly effective as well as safe. The technique may serve as an alternative to multilevel arthrodesis. Implantation of a motion-preserving dynamic stabilization device immediately adjacent to a fused level instead of extending a rigid construct may reduce the subsequent development of adjacent-segment disease in this patient population.

## Introduction

Arthrodesis has been commonly utilized for symptomatic degenerative conditions of the lumbar spine, such as spinal stenosis, degenerative disc disease, and spondylolisthesis. The operation has shown satisfactory clinical results and can be especially therapeutic when coupled with nerve decompression for lower back pain relief [1]. Furthermore, procedural advances have led to a decreased incidence of postoperative immobilization and brace therapy [2]. Unfortunately, studies have shown a high degree of variability in the efficacy of spinal fusion, with revision rates as high as 36% in some instances [1, 3]. Lumbar fusion also carries a significant risk for the development of adjacent segment disease (ASD) with incidence rates reportedly as high as 30%, resulting in increased morbidity and subsequent revision surgery [4]. ASD is thought to be caused by abnormal biomechanical forces imparted by the rigid spine, which advances symptomatic degenerative changes in the immediately adjacent mobile segments [5].

Dynamic stabilization has emerged in recent years as a surgical alternative to lumbar spinal fusion for the treatment of spinal stenosis. Although biomechanical models have shown dynamic stabilization to minimize pressures at, as well as adjacent to, the level of spinal instrumentation, the incidence of ASD after dynamic stabilization remains [6-8]. Still, nerve decompression coupled with instrumented dynamic stabilization may be preferable to arthrodesis since the former can ameliorate neurogenic lower back and leg pain while still allowing for a range of motion in the spine [1]. One lumbar motion preservation device is the Dynesys^®^ Dynamic Stabilization System (Zimmer-Biomet Spine, Denver, CO), which utilizes pedicle screws interconnected with flexible polyethylene terephthalate (PET) cords and polycarbonate urethane spacers in order to preserve motion in the lumbar spine.

The Dynesys Transition Optima™ (DTO) (Zimmer-Biomet Spine, Denver, CO), a unique system, enables hybrid rigid to dynamic stabilization. This transition system allows for arthrodesis of critically unstable vertebrae in combination with the dynamic stabilization of adjacent, moderately degenerated segments [9]. Revision rates ranging from 10% to 34% have been reported with the use of the non-hybrid, traditional Dynesys^®^ system [10-12]. Few studies have reported on the clinical experience with the DTO hybrid system. We previously reported our initial experience with DTO in a 24-patient cohort [13]. With over ten years of experience with dynamic stabilization systems, including DTO, the current study was undertaken to clinically evaluate the DTO system in a long-term fashion.

## Materials and methods

### Demographics

**Figure 1:**
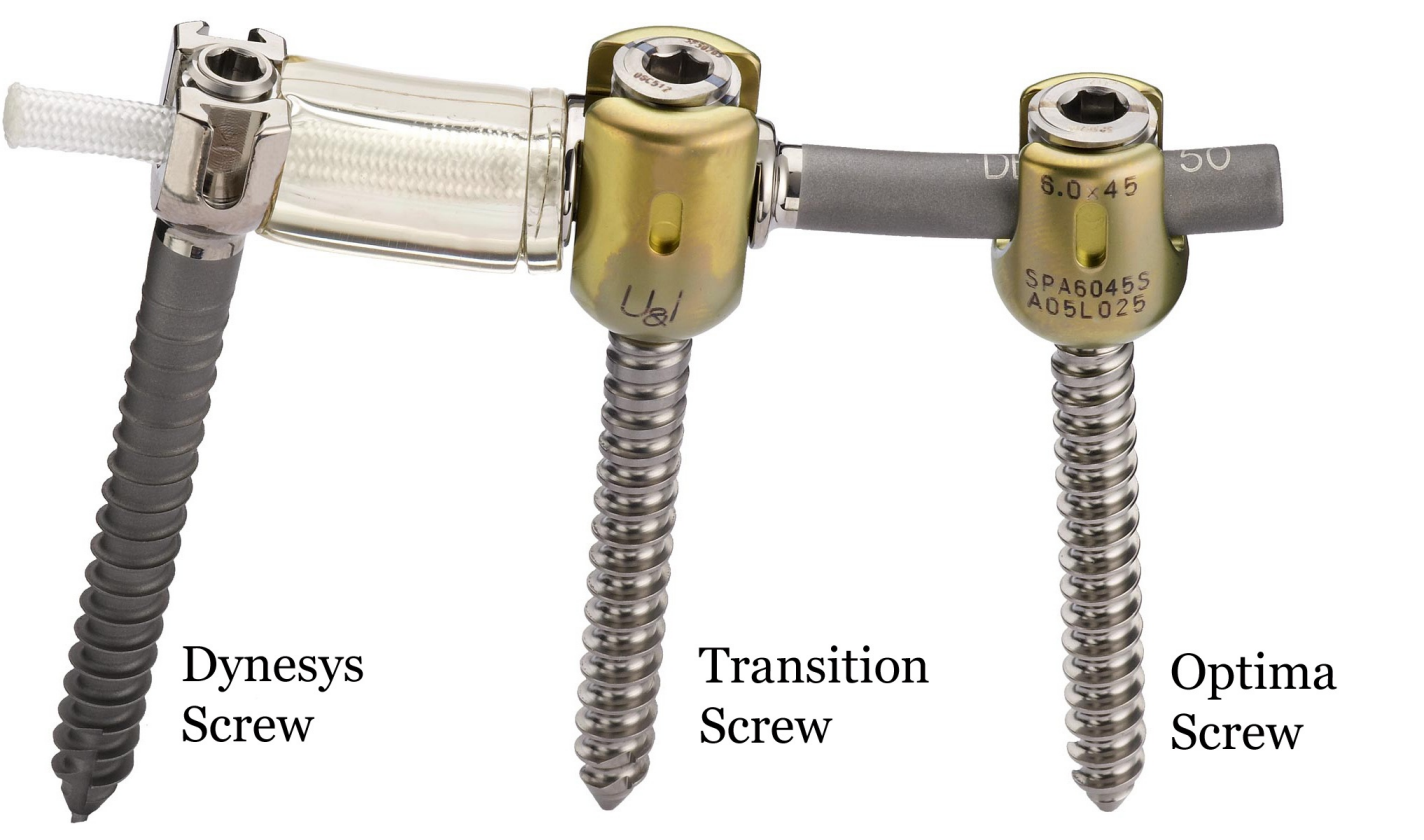
Photograph of the Dynesys Transition Optima (DTO) implant, which is a hybrid construct with a dynamically stabilized segment (on the left) and a rigidly fixated segment (on the right).

**Figure 2:**
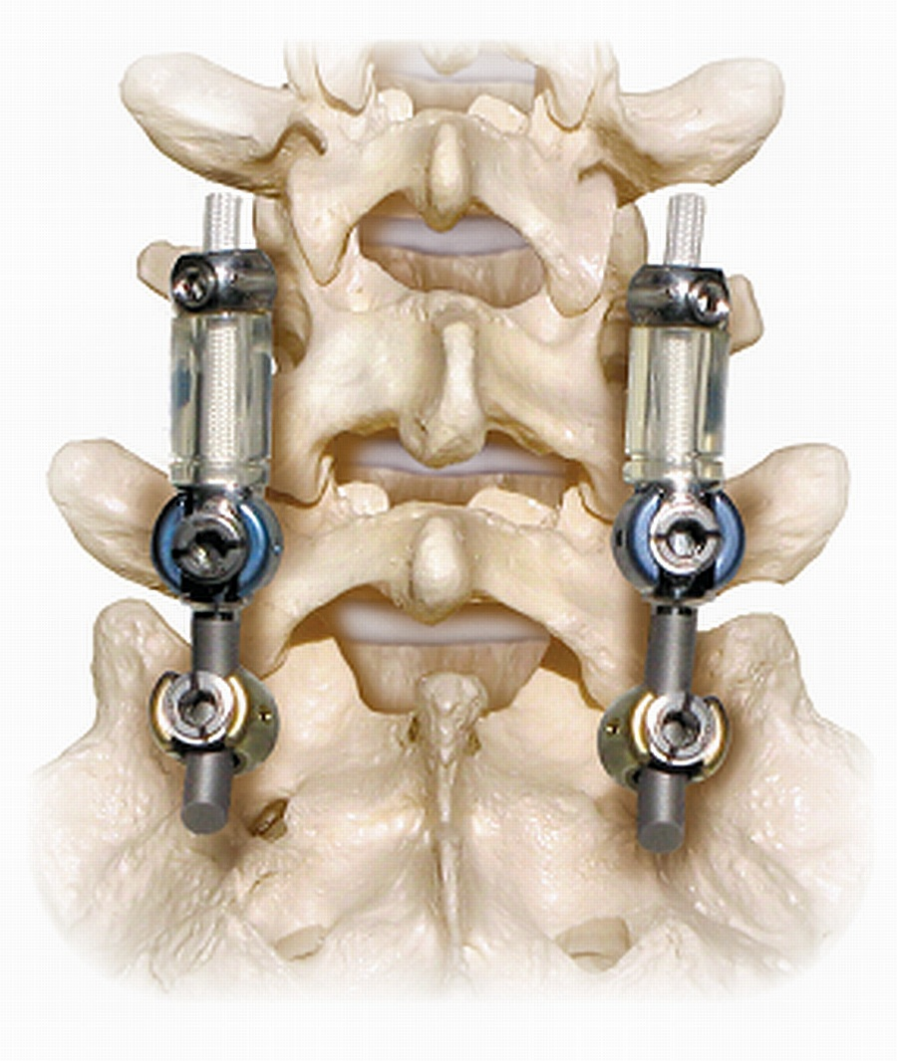
Demonstration of the DTO implant, with a fusion at the L5-S1 level and dynamic stabilization at the L4-5 level.

A total of 66 patients, average age 53 years (range: 25-76 years), underwent lumbar spinal surgery with the DTO hybrid system (Figures 1-2). The median length of follow-up was five years. Selected cases demonstrated radiographical signs of spondylolisthesis, spinal stenosis, degenerative disc disease, and/or disc herniation. Patients were selected for the DTO procedure if they presented with moderate degenerative stenosis adjacent to sections of significant spinal instability in which a rigid fusion was deemed indicated. For the DTO implant, patients were selected if they had a least one level of severe instability that required a fusion with an adjacent symptomatic level demonstrating disc herniation or stenosis without significant instability. The pathology at the rigid level was different than the pathology at the level of dynamic stabilization in all cases. At the rigid level, there was evidence of moderate to severe lateral recess stenosis or facet arthropathy requiring rigid fixation. At the level of dynamic stabilization, there was evidence for only mild to moderate stenosis. All dynamically stabilized levels were rostral to the fused levels. Not all levels of fusion had a spondylolisthesis. All patients experienced clinical symptoms of back pain and neurogenic claudication refractory to non-surgical treatment.

### Surgical technique

All surgeries were performed by the senior author after obtaining informed consent from each subject. The DTO system was utilized to achieve a solid arthrodesis at the level of rigid instrumentation and was not being used as an adjunct to fusion at the level that was dynamically stabilized. Surgeries were performed with the patient in a prone position under general anesthesia using midline lumbar incisions. As necessary, surgical decompression of the cauda equina and nerve roots was accomplished via discectomy, laminectomy, and/or facetectomy before instrumentation. A laminectomy was performed at the fused level in all cases. A full laminectomy was not performed at all levels of dynamic stabilization. Bilateral facetectomies were only performed in cases in which bilateral stenosis was present. At the level of dynamic stabilization, a discectomy was performed if there was a large disc herniation present.

The DTO implant combines the Dynesys Neutralization System and the Optima Spinal System. The Dynesys implant consists of polyethylene-terephthalate (PET) cords, polycarbonate urethane spacers, and titanium alloy pedicle screws. The spacers are placed bilaterally between the pedicle screw heads to withstand compressive loads. The cords are run through the hollow core of the spacers and stabilize the construct by a tensile preload. The Dynesys/DTO system is currently approved by the United States Food and Drug Administration (US FDA) as an adjunct to spinal fusion. Its use for this study was in an off-label manner.

Patients are placed in a prone position with care taken to preserve the natural lordosis of the lumbar spine. A standard midline lumbar incision over the spinous processes of the vertebrae is used with a bilateral subperiosteal muscle separation approach. All pedicle screws are placed under real-time fluoroscopy to ensure appropriate placement. Correct screw placement is absolutely necessary for optimal functioning of the Dynesys system. A spacer template is used to determine the correct position of the screws. Pedicle screws are placed lateral to the facet joints to avoid facet disruption. A spacer template is used to determine the correct position of the screws relative to one another, to allow for optimal placement of the spacer device. A pedicle probe is used to create a channel for the screw under fluoroscopic guidance. Every attempt is made to use the largest diameter and longest screw possible according to the patient’s anatomy. The pedicle screw is advanced in a lateral-to-medial trajectory until the head of the screw is at the synovium of the joint.

Following pedicle screw placement, a pedicle distance gauge is placed between the screw heads in the center of the holes to measure the appropriate spacer length. The distance (spacer length) is measured with a slight distraction force. The PET cord is then advanced through the first screw with at least 10 mm of cord remaining outside the screw head. The anti-torque instrument is placed onto the screw head. The set-screw driver is applied to the screw head and tightened until the torque-limiting driver snaps. The cord is pushed through the appropriately sized spacer, and the spacer is placed against the first screw head. The cord is then inserted through the second screw. The cord guide is placed on the guide pin and screw, and the cord-tensioning instrument is placed on top of the cord guide. Care is taken to keep the cord, spacer, and screws in absolute proper alignment.

The cord-tensioning instrument is used to pull the spacer into proper position. The set-screw is inserted into the cord guide using the set-screw starter. The set-screw driver is attached to the torque-limiting driver and then engaged with the set-screw. While maintaining tension on the cord, the set-screw is tightened until the torque-limiting driver snaps. The procedure is repeated for the contralateral side and can be repeated for adjacent levels if needed. When the system is fully tensioned, the cords are cut, leaving at least 10 mm of cord extending from the screw heads.

Dynesys screws (6.25 mm in diameter and 45 to 55 mm in length) were placed under real-time fluoroscopy in a lateral-to-medial trajectory at the base of the transverse process as to avoid facet damage [14]. Longer screws were utilized when possible, depending on the patient’s vertebral anatomy. The spacers were placed bilaterally between the screws to resist compressive forces and threaded with the PET cords for tensile strength [15-16].

Instrumented fusion was performed by placing screws bilaterally into the pedicles with a bony autograft between segments. Optima screws (6.0 to 7.0 mm in diameter and 45 to 55 mm in length) were attached posteriorly with a standard 6 mm titanium rod [14]. The Dynesys dynamic stabilization system and Optima rigid fixation system were separated by a transition pedicle screw.

### Data collection

The authors independently reviewed the prospectively maintained database of all dynamic stabilization procedures performed between 2005 and 2015 at the University of Pittsburgh Medical Center. Medical records were reviewed for procedure characterizing information, such as underlying diagnoses, prior surgeries, frequencies of interbody fusion and decompression, levels of instrumentation, infectious and non-infectious complications, and subsequent spinal surgeries. Approval for the study was received from the University of Pittsburgh’s institutional review board (protocol #PRO07060042.

## Results

**Figure 3:**
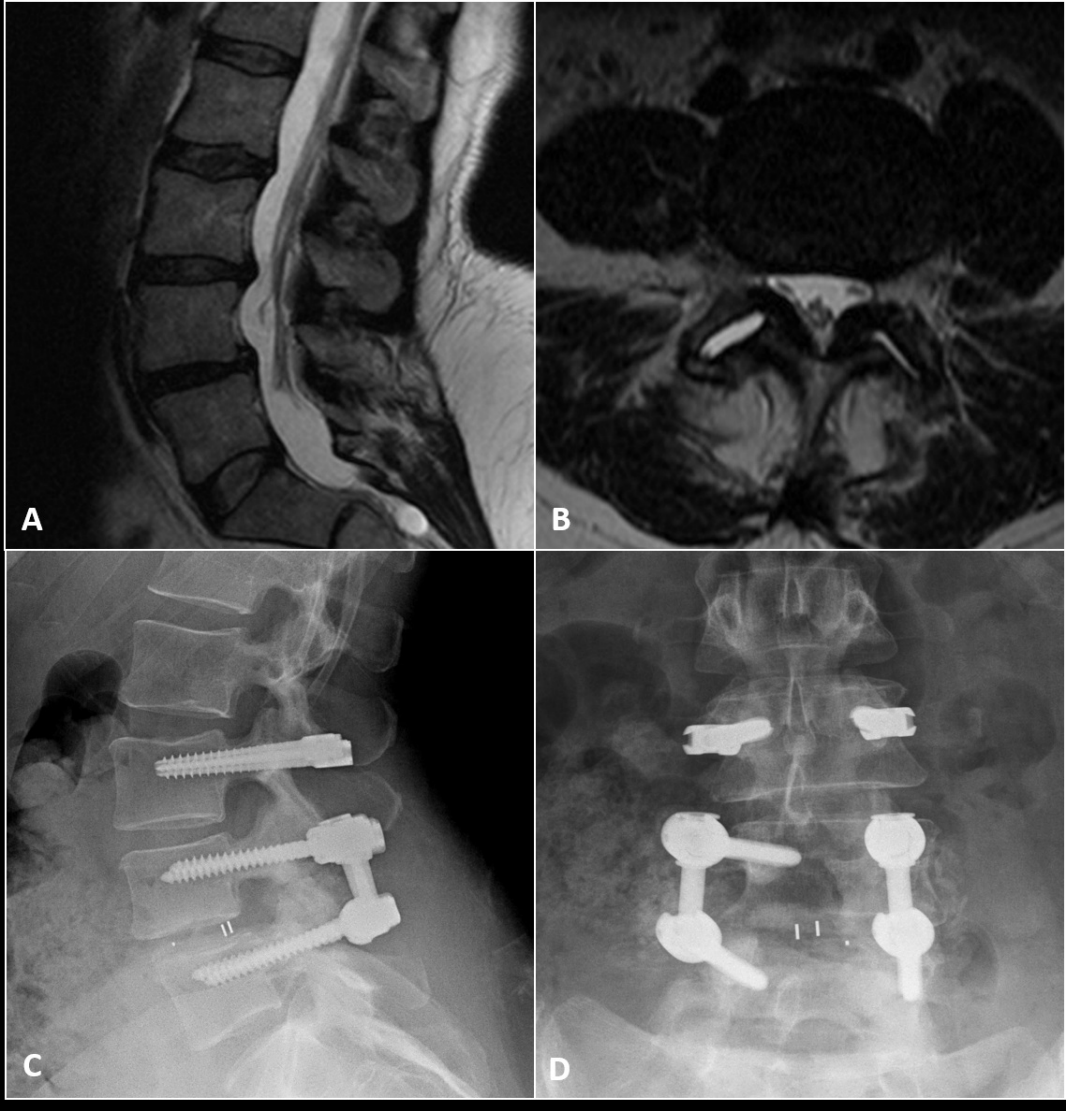
Case example of a 54-year-old woman with disabling low back and leg pain in whom nonsurgical therapy had failed. A: Preoperative sagittal T2-weighted MR image reveals a Grade I spondylolisthesis of L4-5 with a large L3-4 disc herniation. B: Preoperative axial T2-weighted MR image through the L4-5 disc space reveals bilateral facet disruption. It was elected to perform an L4-5 arthrodesis and L3-4 dynamic stabilization procedure. C: A postoperative lateral radiograph demonstrating the hybrid construct with a dynamically stabilized segment (L3-4) above a rigid fused segment (L5-S1). An interbody spacer was placed at L4-5. In all cases, a lordotic rod was implanted in order to preserve and/or restore normal lumbar lordosis. Every attempt was made when implanting the bumper to lock the patient into a lordotic posture. D: Postoperative AP radiograph demonstrating the DTO hybrid construct.

Between 2005 and 2015, 66 consecutive patients underwent implantation with the DTO system. Figure 3 demonstrates a typical case example. Forty patients (61%) who underwent DTO instrumentation had at least one previous lumbar surgery at the same level. Prior levels of surgery were most frequent in the lower lumbar segments, namely L5-S1 (n = 30, 45%). The primary indications for surgery were primary degenerative disc disease (n = 52, 79%) and failed back surgery syndrome (n = 14, 21%). The most common stabilized and fused segments were L3-L4 (n = 37, 56%) and L5-S1 (n = 33, 50%), respectively. Thirty-eight patients (58%) underwent decompression at the dynamically stabilized level, and 57 patients (86%) had an interbody device placed at the level of arthrodesis. Interbody devices were placed in order to increase the likelihood of arthrodesis. In cases of high-grade spondylolisthesis or extensive degree of epidural fibrosis, interbody devices could not be safely implanted. Patient characteristics are summarized in Table 1.

**Table 1:** Summary of Patient Cohort

Variable	Number of Patients n = 66 (%)
Demographics	
Mean Age (Range)	53 (25-76)
Sex, Male	35 (53.0)
Indication	
Primary Degenerative Disc Disease	52 (78.8)
Failed Back Surgery Syndrome	14 (21.2)
Prior Spine Surgery	
Total Patients	40 (60.6)
Arthrodesis	15 (22.7)
Discectomy	15 (22.7)
Laminectomy	8 (12.1)
Microcervical Discectomy	4 (6.1)
Pedicle Screw Implant	2 (3.0)
Dynamic Stabilization	1 (1.5)
Dynamically Stabilized Segments	
L1-2	1 (1.5)
L2-3	11 (16.7)
L3-4	37 (56.1)
L4-5	22 (33.3)
L5-S1	4 (6.1)
Fused Segments	
L2-L3	6 (9.1)
L3-4	10 (15.2)
L4-5	27 (40.9)
L5-S1	33 (50.0)
Procedures Performed	
Interbody Fusion	57 (86.4)
Decompression	38 (57.6)

During the follow-up period, 21 patients (32%) required additional lumbar spine surgery after being implanted with DTO. There were 10 patients (15%) who underwent conversion of the dynamic stabilization portion of their DTO instrumentation to rigid spinal arthrodesis for new symptoms or persistence of their preoperative symptomatology. The indications for conversion of dynamic stabilization to fusion are included in Table 2. The indications for revision surgery included progression of spinal stenosis, the persistence of lower back pain, and disc herniation at the level of dynamic stabilization. Postoperatively, three (4%) interbody cage migrations and one (1%) broken screw were observed. This broken screw was at the level of the transition from rod to dynamic stabilization. A single interbody cage migration required surgical revision. Pseudoarthrosis requiring surgical revision occurred in only a single patient. Two patients (3%) developed wound infections.

**Table 2:** Summary of Postoperative Complications *One patient underwent spinal fusion from T11-S1 postoperatively

Variable	Number of Patients n = 66 (%)
Subsequent Spine Surgery	21 (31.2)
Interbody Cage Migration	3 (4.5)
Infection	2 (3.0)
Screw Breakage	1 (1.5)
Indications for Conversion to Fusion	
Total Converted Patients	10 (15.2)
Progressive Spinal Stenosis	5 (9.0)
Disc Herniation	2 (3.0)
Continued Lower Back Pain	2 (3.0)
Pseudoarthrosis	1 (1.5)
Progressive Spondylolisthesis	1 (1.5)
Symptomatic Cage Migration	1 (1.5)
Broken Screw Requiring Revision	1 (1.5)
Levels for Conversion Surgery	
T11-L1*	1 (1.5)
L1-L2	2 (3.0)
L2-L3	4 (6.1)
L3-L4	6 (9.1)
L4-L5	6 (9.1)
L5-S1	2 (3.0)

## Discussion

In the past 20 years, the range of spine stabilization therapies has been augmented by the advent of motion preservation systems that attempt to decrease the incidence of ASD after rigid fixation [17]. These systems have shown satisfactory results in terms of pain relief, safety, quality of life, and motion preservation [18-20]. By preserving physiologic motion of the spine, dynamic fixators mitigate hypermobility and the disruptive biomechanical forces observed in adjacent segments after arthrodesis [6, 18]. Despite these advantages, the role of non-fusion systems in the context of ASD remains ambiguous. For instance, two recent meta-analyses suggested that non-fusion stabilization of the spine protects against subsequent ASD [21-22]. In contrast to these studies, St-Pierre, et al. reported that new-onset clinical ASD (29%) after Dynesys stabilization was higher than ASD observed after arthrodesis (5.2 - 16.5%) at five-year follow-up [23].

The more recent development of the DTO hybrid system can be utilized for the rigid stabilization of multilevel spinal degeneration while allowing for a limited degree of motion in the adjacent dynamically stabilized segments. DTO implantation is a good alternative to rigid fixation in the carefully selected patient, being ideal for those patients with critical spinal instability and adjacent level pathology of lesser severity that requires decompression but not necessarily rigid fixation. Overall, data regarding this hybrid system is limited, but some small retrospective reviews indicate that DTO implants can alleviate back pain with ASD reported in 10.0% and 12.5% of patients at five-year and mean eight-month follow-ups, respectively [13-14]. Lee, et al. recently compared ASD development between rigid fixation and hybrid fusion to dynamic stabilization at two-year follow-up and found that hybrid systems delayed, but did not prevent, ASD [24]. In another study of 41 patients treated with hybrid stabilization, Formica, et al. found no significant degenerative changes in adjacent segments at two-year follow-up [25].

There is an abundance of literature regarding traditional, non-hybrid Dynesys instrumentation and its associated complications [26-27]. Infection rates have been generally well controlled during these procedures [12, 27]. Our initial experience with DTO reported a wound infection rate of 8% [13]. In the current study, we report an improved infection rate of only 3%. This finding is also comparable to infection rates associated with instrumented arthrodesis, which varies from 1 to 11% [28]. Revision rates following DTO implantation are not well documented in the spine literature, but re-intervention after non-hybrid Dynesys instrumentation has been reported to be between 10 and 34% [10-12, 27]. Grob, et al. conducted a two-year follow-up in 31 patients that underwent Dynesys stabilization. Of the implants, two required conversion to formal arthrodesis, one became infected, one required the implantation of an intrathecal pump, and two exhibited signs of screw loosening [12]. More recently, Baioni, et al. reported no cases of implant breakage or screw loosening in five-year follow-up with a 30 patient cohort of DTO implants [14]. In our previous analysis, we reported treatment failure in 12.5% of patients implanted with DTO [13].

The overall incidence of hardware failure was exceedingly low. In the current study, we found only a single case of subsequent screw breakage during follow-up imaging. However, three cases of interbody cage migration were observed on follow-up imaging. This incidence of interbody cage migration is somewhat higher than our rate in standard arthrodesis cases. While a small sample size may reflect the error of undersizing the cage in these three cases, there may also be a relationship to the fact that the rigid rod is directly connected to a motion preservation system. Further monitoring will be required to ensure that the rates of arthrodesis with the DTO system are equivalent to those of standard methods.

## Conclusions

This study represents a long-term cohort evaluation of a unique hybrid lumbar dynamic stabilization to fusion system. The DTO system was found to be a safe and viable technology that allows for the coupling of arthrodesis with dynamic stabilization at adjacent levels in the lumbar spine. The incidence of wound infection and implant failure is no higher than with conventional arthrodesis systems. There were no cases of dynamic stabilization implant failure or screw loosening. The present series supports the efficacy, safety, and reliability of this hybrid posterior fixation system as an alternative to multilevel lumbar arthrodesis. Careful patient selection is still required for those patients who would benefit from arthrodesis rather than dynamic stabilization at adjacent levels of pathology.
